# Tissue culture and next-generation sequencing: A combined approach for detecting yam (*Dioscorea* spp.) viruses^☆^

**DOI:** 10.1016/j.pmpp.2018.06.003

**Published:** 2019-01

**Authors:** Moritz Bömer, Ajith I. Rathnayake, Paul Visendi, Steven O. Sewe, Juan Paolo A. Sicat, Gonçalo Silva, P. Lava Kumar, Susan E. Seal

**Affiliations:** a Natural Resources Institute, University of Greenwich, Central Avenue, Chatham Maritime, Kent, ME4 4TB, UK; b International Institute of Tropical Agriculture (IITA), Oyo Road, PMB 5320, Ibadan, Nigeria

**Keywords:** *In vitro* culture, RNA-Seq, *Dioscorea* spp., *Badnavirus*, *Yam mosaic virus*, *Yam mosaic virus*, YMV, *Yam mild mosaic virus*, YMMV, *Cucumber mosaic virus*, CMV, *Dioscorea bacilliform virus*, DBV, endogenous pararetroviruses, EPRV

## Abstract

*In vitro* culture offers many advantages for yam germplasm conservation, propagation and international distribution. However, low virus titres in the generated tissues pose a challenge for reliable virus detection, which makes it difficult to ensure that planting material is virus-free. In this study, we evaluated next-generation sequencing (NGS) for virus detection following yam propagation using a robust tissue culture methodology. We detected and assembled the genomes of novel isolates of already characterised viral species of the genera *Badnavirus* and *Potyvirus*, confirming the utility of NGS in diagnosing yam viruses and contributing towards the safe distribution of germplasm.

## Highlights

• Robust tissue culture method boosts *in vitro* growth of *D. alata* and *D. rotundata*.
• Next-generation sequencing detected known and novel viruses in yam tissue culture.
• Sequencing results enabled phylogenetic analysis of three novel yam virus isolates.

## 1 Introduction

Yam (*Dioscorea* spp. of family Dioscoreaceae) is a multi-species crop that generally produces large, starchy tubers used as a popular food staple in Africa and Asia. In West and Central Africa, yams play a principal role in food and nutrition security and income generation for more than 60 million people and are important in cultural life [[1], [2], [3], [4]]. The major cultivated yam species globally are *D. alata*, *D. bulbifera*, *D. cayenensis*, *D. esculenta*, *D. opposita-japonica*, *D. nummularia*, *D. pentaphylla*, *D. rotundata*, and *D. trifida* [5]. The species *D. cayenensis* and *D. rotundata* are indigenous to West Africa, where they are the two most important yam species in terms of yield produced. In contrast, *D. alata* is of Asiatic origin and is the most globally widespread species of yam [1]. Yam is mainly cultivated by smallholder farmers, and the ‘yam belt’ stretching across Benin, Ivory Coast, Ghana, Nigeria, and Togo in West Africa is the world's dominant zone for yam production. According to reports of the International Institute of Tropical Agriculture (IITA), the demand for this food security crop is always higher than the actual supply and, with an increasing population, that trend is expected to continue [1].

Yams are annual or perennial vines and climbers with underground tubers [6]. Cultivated yams are generally propagated vegetatively using their tubers, which leads to the perpetuation and accumulation of tuber-borne pathogens, particularly viruses [7]. Virus species belonging to at least six different genera infect yams in West Africa [[7], [8], [9]], causing severe impacts on tuber yield and quality as well as impeding yam germplasm movement. *Yam mosaic virus* (YMV; genus *Potyvirus*), *Yam mild mosaic virus* (YMMV; genus *Potyvirus*), *Cucumber mosaic virus* (CMV; genus *Cucumovirus*), and several species of *Dioscorea*-infecting badnaviruses have been reported to be widespread across the ‘yam belt’ in West Africa [[10], [11], [12], [13], [14]]; YMV is often described as the most economically important of these. The first and only complete YMV genome (an Ivory Coast isolate) was reported by Aleman et al. [15] in 1996. YMV was first identified in *D. cayenensis* by Thouvenel and Fauquet in 1979 [16] and has a single-stranded, positive-sense RNA genome of 9608 nucleotides in length that is encapsidated in flexuous filamentous particles. YMV is transmitted horizontally by aphids in a non-persistent manner as well as by mechanical inoculation. It is also transmitted vertically by vegetative propagation of infected plant material [15,17]. YMV infection is associated with a range of symptoms, including mosaic, mottling, green vein banding, leaf deformation, and stunted growth, leading to reduced tuber yield.

Badnaviruses are plant pararetroviruses (family *Caulimoviridae*, genus *Badnavirus*) that have emerged as serious pathogens infecting a wide range of tropical and subtropical crops; these include banana, black pepper, cacao, citrus, sugarcane, taro, and yam [18]. Badnaviruses have bacilliform-shaped virions that are uniformly 30 nm in width, have a modal particle length of 130 nm, and contain a single molecule of non-covalently closed circular double-stranded DNA in the range of 7.2–9.2 kbp with each strand of the genome having a single discontinuity [19]. Badnavirus replication involves the transcription of a single, greater-than-genome length, terminally redundant pre-genomic RNA, which serves as a polycistronic mRNA for translation of the genome's three open reading frames (ORFs) and is used as the template for DNA synthesis in the cytoplasm [19]. Badnaviruses transport their DNA into the host nucleus for transcription, and random integration of the viral DNA into the host genome may occur through illegitimate recombination or during the repair of DNA breaks [20,21]. The genus *Badnavirus* is the most diverse within the family *Caulimoviridae*, and the genetic and serological diversity of its members, along with the occurrence of integrated viral counterparts termed endogenous pararetroviruses (EPRV) in the genomes of its hosts, complicate the development of reliable diagnostic tools based on DNA detection [[22], [23], [24], [25]].

*Dioscorea* bacilliform viruses (DBVs) are members of the *Badnavirus* genus and can accumulate across yam cultivation cycles. DBVs present a serious threat to the safe movement of yam germplasm because of their high prevalence and extreme heterogeneity [24,[26], [27], [28], [29], [30]]. Diverse badnaviruses in single and mixed infections have been identified in West African yam germplasm [27,31,32], and *D. cayenensis-rotundata* genomes have been shown to contain endogenous *Dioscorea* bacilliform viruses (eDBVs) as integrated forms of these viruses [14,25,27,33]. To date, eight distinct DBV genomes have been completely sequenced: Dioscorea bacilliform AL virus (DBALV), Dioscorea bacilliform AL virus 2 (DBALV2), Dioscorea bacilliform ES virus (DBESV), Dioscorea bacilliform RT virus 1 (DBRTV1), Dioscorea bacilliform RT virus 2 (DBRTV2), Dioscorea bacilliform RT virus 3 (DBRTV3), Dioscorea bacilliform TR virus (DBTRV), and Dioscorea bacilliform SN virus (DBSNV) [27,31,32,[34], [35], [36]]. Phylogenetic analysis based on these genome sequences together with several hundred partial badnavirus sequences led to proposals that at least 15 badnavirus species are associated with yam [11,14,24,[26], [27], [28], [29], [30],^32^,^33^,^37^].

The only effective method of controlling the above viral diseases is to use virus-free (‘clean’) planting material. The scarcity and associated high expense of such material has been identified as one of the most important factors limiting yam production in West Africa [3]. Yam production has historically been hindered by the low rate of multiplication achieved by conventional yam propagation methods (e.g. seed tubers), which are slow and inadequate for rapid multiplication [38]. Plant tissue culture techniques have the potential to overcome some limitations of conventional propagation methods in yams. Studies by Aighewi et al. and IITA showed that aeroponics and temporary immersion bioreactor systems (TIBs) produce improved multiplication rates and higher-quality planting material compared with techniques using ware and seed tubers (including the minisett technique) or vine cuttings [39,40]. These *in vitro* culture techniques can potentially deliver high-quality, clean, clonal plant material and may therefore represent a sustainable solution for the rapid production of pathogen-free planting material [39,41].

Yam tissue culture is currently used in the exchange of genetic material between countries, and in scientific research, such as rapid increase of planting material for phenotyping to various biotic and abiotic stresses, in the efficient transformation of yam lines, for the production of virus-free yam lines, and other applications. Techniques and applications for the *in vitro* propagation of members of the genus *Dioscorea* have been widely researched [38,[41], [42], [43], [44], [45], [46], [47]], and revealed that *in vitro* propagation and virus indexing for the two most important yam species, *D. alata* and *D. rotundata*, still need improvements.

Several serological and nucleic acid-based methods, such as enzyme-linked immunosorbent assay (ELISA), immunocapture reverse transcription-PCR (IC-RT-PCR), RT-PCR, reverse-transcription recombinase polymerase amplification (RT-RPA), closed-tube reverse transcription loop-mediated isothermal (CT-RT-LAMP), and rolling circle amplification (RCA), have been used in indexing known yam viruses and also to characterise new yam potyviruses and badnaviruses [[48], [49], [50], [51], [52], [53]]. Next-generation sequencing (NGS) methods are increasingly being employed in the discovery and sequencing of new plant viral genomes [54,55]. Whereas established plant pathogen diagnostic strategies such as ELISA and PCR target specific species, the massively parallel approaches of NGS generate high-throughput data that can be directly analysed for both known and unknown pathogens without the need for prior knowledge of the target sequences [54]. Consequently, NGS has potential as a robust and sensitive detection method for confirmation of virus-free material. However, in their review, Blawid et al. [54] point out that it is necessary to establish sensitive and robust assembly pipelines targeting small viral genomes and ones characterised by low identities to known viral sequences.

Yam is still an understudied ‘orphan’ crop that demands much more research attention. NGS and bioinformatics tools promise to help fill the knowledge gap around yam genomics and yam viral pathogens. Tamiru et al. [56] recently reported the whole genome sequencing of *D. rotundata*; this will serve as a springboard towards gene discovery and ultimately genetic improvement of this neglected staple crop. In this study, we describe a method for identifying infected planting material using the combination of robust *in vitro* propagation of *D. alata* and *D. rotundata* and NGS-based virus detection in yam tissue culture using Illumina HiSeq4000 RNA sequencing.

## 2 Material and methods

### 2.1 Plant material

**Fig. 1:**
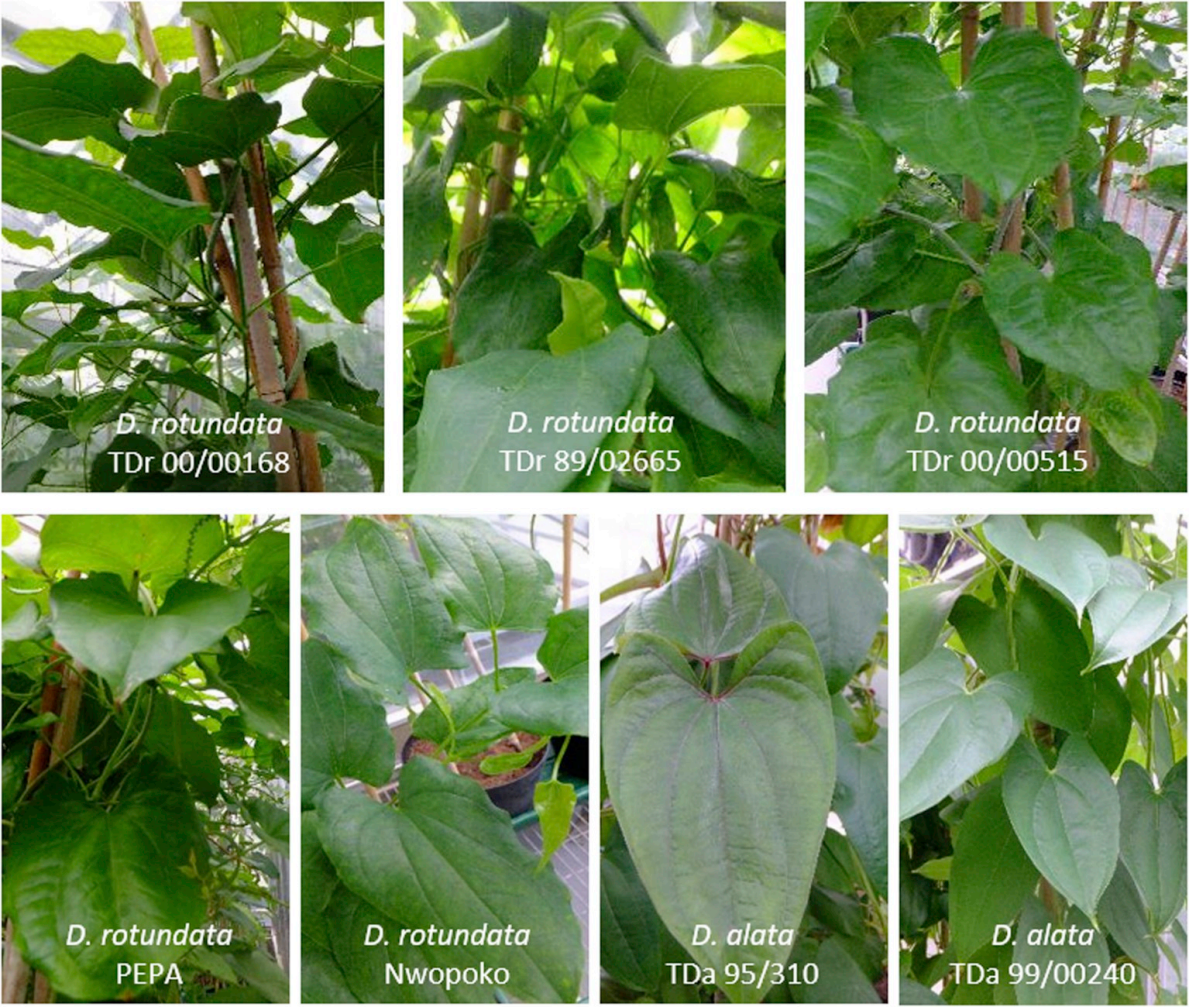
Breeding lines and landraces of *D. alata* and *D. rotundata* used in this study. Yam plants were in an active growth stage when they were used as a source of explant material for the establishment of a robust *in vitro* propagation protocol.

Yam breeding lines and landraces of *D. alata* (*n* = 2) and *D. rotundata* (*n* = 6) used in this study were provided by the IITA (Ibadan, Nigeria). Tubers were known to be infected by YMV and badnaviruses as tested by conventional RT-PCR and PCR at IITA using generic primers respectively, but the precise status of species and occurrence of any other virus was not known. Tubers were grown in a quarantine aphid-proof glasshouse at the Natural Resources Institute (NRI, Chatham, UK), as described by Mumford and Seal [49]. Actively growing plants of the *D. rotundata* breeding lines (TDr 00/00515, TDr 00/00168, and TDr 89/02665) and landraces (Nwopoko and Pepa), and the *D. alata* breeding lines (TDa 95/310 and TDa 99/00240) (Fig. 1), were used as a source of explant material for *in vitro* propagation experiments. *D. rotundata* landrace (cv. Makakusa) from Nigeria showing viral symptoms was chosen for the experiments involving NGS-based virus discovery.

### 2.2 Yam *in vitro* culture

Vine cuttings from a single plant of each genotype, usually containing one to three nodes, were trimmed to 5–8 cm and leaves removed. Each cutting was placed in a 1-l bottle half-filled with tap water. The cuttings were washed twice with tap water through vigorous shaking by hand. The explant materials were then immersed in 70% v/v ethanol for 3–5 s and immediately transferred to 250 ml of a sterilisation solution consisting of 5% w/v sodium hypochlorite (NaClO) with 1–2 drops of Tween-20. Bottles containing explant materials and the sterilisation solution were incubated with a SF1 flask shaker (Stuart Scientific, UK) for 20 min at 500 oscillations/min. The sterilisation solution was decanted in a laminar flow cabinet under sterile conditions, and the cuttings were rinsed three times with sterilised deionised double-distilled water. Two different *in vitro* culture media compositions (M1 and M2) were tested for their suitability for the *in vitro* propagation of selected yam accessions (Table 1). The effects on plant growth of both media with and without activated charcoal (AC) were tested.

**Table 1:** Yam *in vitro* culture media compositions tested.

Chemical	M1^a^	M2^b^
MS basal medium (M5519)	4.4 g/l	–
MS basal medium with Gamborg vitamins (M0404)	–	4.4 g/l
Sucrose	30 g/l	30 g/l
Kinetin	0.5 mg/l	–
Cysteine	20 mg/l	–
6-Benzylaminopurine (BAP)	–	0.05 mg/l
Naphthaleneacetic acid (NAA)	–	0.02 mg/l
Ascorbic acid	–	25 mg/l

a Previously described in IITA yam *in vitro* genebanking manual (www.iita.org/wp-content/uploads/2017/Yam_in_vitro_genebanking.pdf) with slight modifications.

b Previously reported by Nyaboga et al. [47].

Both media compositions were adjusted to pH 5.8 using 0.1 M NaOH solution and then supplemented with 2 ml/l of plant preservative mixture (Plant Cell Technology, USA) and 2 g/l Phytagel™ (Sigma-Aldrich, UK). Half of the culture tubes for each medium were supplemented with 0.2% w/v AC. Of media, 8 ml was dispensed into culture tubes (specimen tubes soda glass poly stopper 100 × 25 mm, G050/30, Fisher brand, USA) and autoclaved. All chemicals were obtained from Sigma-Aldrich UK, unless otherwise indicated.

Under sterile conditions, surface-sterilised explant materials were sized to 1.0–1.5 cm length, each containing a single node with axillary buds, and placed in culture tubes containing one of the two culture media. Culture tubes were placed in a plant growth incubation room where the temperature was maintained at 25 ± 1 °C and the light was provided by cool white fluorescent lamps with 30–50 μmol/(m^2^·s) for a 16-h photoperiod. The fresh weight of the plantlets was recorded after ten weeks by removing the plantlets from the tubes. The data collected on fresh weight of 145 individual tissue culture tubes (Table S1) were analysed for statistical significance using analysis of variance (ANOVA). Post hoc Tukey HSD tests were performed for multiple comparisons. The statistical analysis was performed using the R statistical software package [57].

Following the establishment of a robust *in vitro* propagation protocol for *D. alata* and *D. rotundata* germplasm, all yam material grown at NRI was conserved in M2 media and culture tubes placed in an A1000 tissue culture chamber (Conviron, UK) maintained at 28 °C and 50% humidity and with light provided by 21 W T5/840 cool white fluorescent lamps with 30–50 μmol/(m^2^·s) for a 16-h photoperiod.

### 2.3 RNA extraction for NGS

Tissue-cultured plants (pool of three tissue culture tubes) of *D. rotundata* (cv. Makakusa) grown *in vitro* for six weeks were used for RNA extraction. Total RNA was extracted from leaf tissues using a modified cetyltrimethyl ammonium bromide (CTAB) method combined with the RNeasy Plant Mini Kit (Qiagen GmbH, Germany). Briefly, 100 mg of leaf tissue snap-frozen in liquid nitrogen was ground in gauge bags (10 cm × 15 cm) (Polybags Ltd, UK) until it became a smooth paste. Pre-warmed (1 ml) CTAB extraction buffer (2% w/v CTAB, 100 mM Tris-HCl, pH 8.0, 20 mM EDTA, 1.4 M NaCl, and 1% v/v β-mercaptoethanol) was added immediately and the tissue was further ground. Plant extract (600 μl) was transferred into a sterile microcentrifuge tube. The tube was briefly vortexed and then incubated at 60 °C for 10 min, mixing the samples by inversion every 2 min. Samples were then allowed to cool to room temperature and an equal volume of phenol:chloroform:isoamyl alcohol (25:24:1) was added. Samples were mixed vigorously by inverting approximately 50 times, followed by centrifugation at 15,800 g for 10 min. The supernatant (400 μl) was transferred into a new sterile microcentrifuge tube to which an equal amount of 100% molecular grade ethanol was added. Samples were mixed, and the mixtures were immediately transferred to RNeasy mini spin columns supplied in 2-ml collection tubes provided with the RNeasy Plant Mini kit. From this step until the elution of the RNA, the RNeasy Plant Mini Kit manufacturer protocol was followed.

### 2.4 RNA library preparation and NGS analysis

Total RNA concentrations and purities were analysed using a NanoDrop 2000 spectrophotometer (Thermo Scientific, UK). High-quality total RNA samples showing 260/280 nm ratios above 2.0 and 260/230 nm ratios above 2.0 were selected and further analysed using the Agilent 2100 Bioanalyzer (Agilent Technologies, UK) to check their RNA integrity number (RIN). RNA samples with RIN values > 7.5 were sent to the Earlham Institute (Norwich, UK) for high-throughput (HT) RNA library construction and Illumina RNA sequencing. The cDNA libraries (HT, non-directional) were constructed using the Illumina TruSeq RNA library kit starting with 3–5 μg of total RNA as input. Ten HT RNA libraries were sequenced on one lane of the Illumina HiSeq4000 platform including one HT library derived from Makakusa RNA. More than 38 million 150-bp paired end reads were generated for the *D. rotundata* cv. Makakusa RNA sample. RNA-seq reads were quality trimmed using Trimmomatic [58] with default parameters. Trimmed reads were then assembled with Trinity v2.5.1 [59] using default parameters. Assembled transcripts were mapped to a custom-made Basic Local Alignment Search Tool (BLAST) database containing complete YMV and badnavirus genomes downloaded from the National Centre for Biotechnology Information (NCBI) GenBank using Geneious v10.2.3 (Biomatters Ltd., New Zealand) [60]. The database included DBALV (X94578, X94580, X94582, and X94575), DBALV2 (KY827395), DBESV (KY827394), DBRTV1 (KX008574), DBRTV2 (KX008577), DBRTV3 (MF476845), DBTRV (KX430257), DBSNV (DQ822073), and YMV (U42596). Transcripts that matched badnavirus genomes were then extended using the Geneious v10.2.3 iterative assembler with ten iterations.

### 2.5 Virus genome characterisation

The assembled transcripts were used for similarity searches in the NCBI GenBank databases (http://www.ncbi.nlm.nih.gov/genbank/) using BLAST [61]. Full-length genome sequences were further analysed in Geneious v10.2.3 and putative ORFs were identified using the NCBI ORF finder (https://www.ncbi.nlm.nih.gov/orffinder/). Conserved domains of the putative gene products were searched using the NCBI conserved domain tool (http://www.ncbi.nlm.nih.gov/Structure/cdd/wrpsb.cgi). Genome maps were generated using SnapGene^®^ Viewer version 4.1 (from GSL Biotech; available at snapgene.com). Multiple alignments of partial 528-bp reverse transcriptase (RT)-ribonuclease H (RNaseH) badnavirus sequences, of the RT-RNaseH gene used for taxonomic assessment of badnaviruses [19], and alignments of the 1184-bp-long YMV nuclear inclusion B-coat protein 3′-untranslated region (NIb-CP-3′-UTR) according to Bousalem et al. [62], were performed using the CLUSTALW default settings in MEGA7 [63]. Complete badnavirus genomes were aligned using Multiple Alignment using Fast Fourier Transform (MAFFT; http://www.ebi.ac.uk/Tools/msa/mafft/) [64]. Phylogenetic analysis was performed in MEGA7 using maximum-likelihood methods based on the Hasegawa–Kishino–Yano model [65]. The robustness of each tree was determined by generating a bootstrap consensus tree using 1000 replicates. Virus sequences obtained from GenBank were used for comparative analyses and accession numbers are shown in the phylogenetic trees. Recombination analysis was performed using the RDP4 software package with default settings [66] and recently described by Bömer et al. [31] in a study on full-length DBV genomes.

### 2.6 RT-PCR assays and Sanger sequencing

Total RNA was extracted from the leaves of cv. Makakusa tissue culture plants as described above. The presence of YMV was confirmed by RT-PCR in the RNA sample used for RNA-seq using the primer pair YMV-CP-1F (5′-ATCCGGGATGTGGACAATGA-3′) and YMV-UTR-1R (5′-TGGTCCTCCGCCACATCAAA-3′), designed by Mumford and Seal [49]. These primers amplify a 586-bp region comprising the coat protein (CP) gene and the 3′-UTR region and were used in a one-step RT-PCR assay performed as described by Silva et al. [51]. The same one-step RT-PCR conditions were used to confirm the DBRTV3-[2RT]/DBRTV3-[3RT] infection using specific primers designed in this study to amplify the RT-RNaseH (579 bp) region of DBRTV3-[2RT], DBRTV3-[2RT]-579 F (5′-ATGCCATTCGGCCTGAAGA-3′), and DBRTV3-[2RT]-579 R (5′-CCATTTGCACACGCCACC-3′). PCR amplification products were analysed by agarose gel electrophoresis, purified using the GeneJET PCR Purification Kit (Fermentas, UK) and Sanger sequenced by the Source BioScience sequencing service (Nottingham, UK).

## 3 Results

### 3.1 Establishment of a robust *in vitro* propagation methodology for yam germplasm

**Fig. 2:**
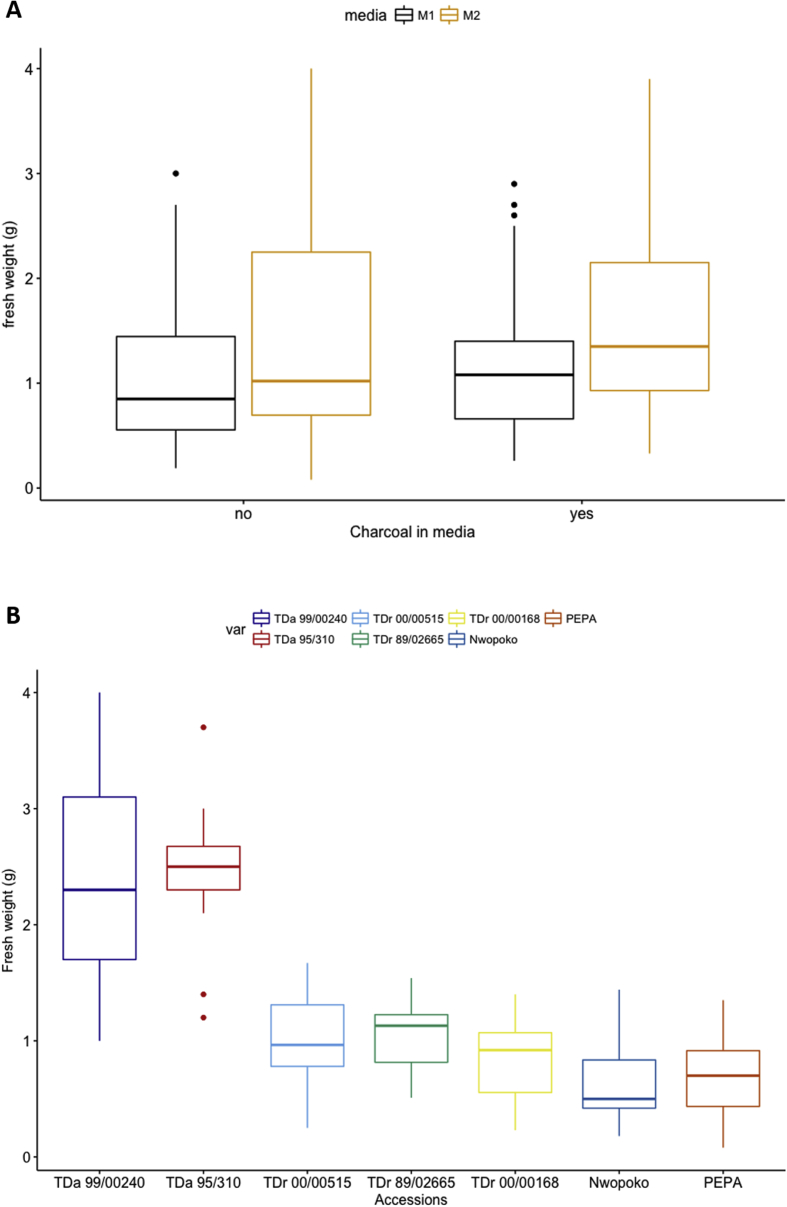
Comparison of fresh weight after 70 days for different yam varieties and culture media. Effect of different *in vitro* culture media compositions and the presence and absence of activated charcoal supplement in the media (**A**) on fresh weight development of seven yam accessions (var) grown as *in vitro* culture for 70 days. Comparison of fresh weight development between individual *D. alata* (TDa) and *D. rotundata* (TDr) accessions (**B**) grown as yam *in vitro* culture. The fresh weight data (g) are expressed as mean ± SE. M1 and M2 denote different *in vitro* culture media compositions described in Table 1.

The effects of the two culture media compositions M1 and M2 and of AC on the fresh weight of yam after 70 days of growth in tissue culture were analysed to establish their impact on *in vitro* propagation of seven accessions of the species *D. alata* and *D. rotundata*. After 70 days in culture, fresh weights of the yam plantlets were recorded and analysed for statistical significance. Both media compositions induced growth of complete plantlets (with shoots and roots) in all yam material tested. The dataset comprised 145 plantlets (Table S1) and was subsequently analysed using three-way ANOVA and post hoc Tukey HSD tests. Analysis revealed a significant effect of the *in vitro* culture media on plant fresh weight (*P* = 0.000198, df = 1, *F* = 14.765) (Fig. 2A). Accessions grown on tissue culture medium M2 had a higher mean fresh weight (1.52 g) than those grown on M1 (1.12 g).

The AC has been reported to improve the growth of some plants in culture, possibly through a combination of its effects on light penetration and its ability to adsorb polyphenolics and other compounds that would otherwise accumulate in the culture medium [67,68]. Here, the effect of media supplemented with 0.2% w/v and without AC on fresh weight development was evaluated. The three-way ANOVA showed a significant effect on fresh weight with the addition of AC to the media (*P* = 0.00104, df = 1, *F* = 11.311) and average fresh weights were increased by 0.2 g (from 1.21 to 1.41 g) (Fig. 2A).

Moreover, the analysis showed that different accessions had significantly different fresh weights (*P* < 0.001, df = 6, *F* = 61.748). The *D. alata* breeding line TDa 95/310 had the highest mean weight (2.4 g), and *D. rotundata* landrace Nwopoko had the lowest (0.6 g) (Fig. 2B). A significant interaction between accession and media was also observed (*P* = 0.0014, df = 6, *F* = 3.880), showing that line TDr 89/02665 performed better on M1 (1.12 g) than M2 (0.99 g), whereas all other tested lines developed higher mean fresh weights when incubated on M2 (Fig. S1). The biggest difference in fresh weight between M1 and M2 was observed in TDa 99/00240. While fresh weights of tissue cultures differed as a function of media and accession, the significant interaction between media and accession suggests that *in vitro* propagation methods specific to an accession could be developed. The *D. alata* accessions TDa 99/00240 and TDa 95/310 developed more fresh weight than *D. rotundata* material. In summary, tissue culture media M2 induced higher mean fresh weights than M1 and hence can be described as a robust yam tissue culture media composition for the *in vitro* multiplication of *D. alata* and *D. rotundata*.

### 3.2 NGS reveals virus infections in yam tissue culture plantlets

**Fig. 3:**
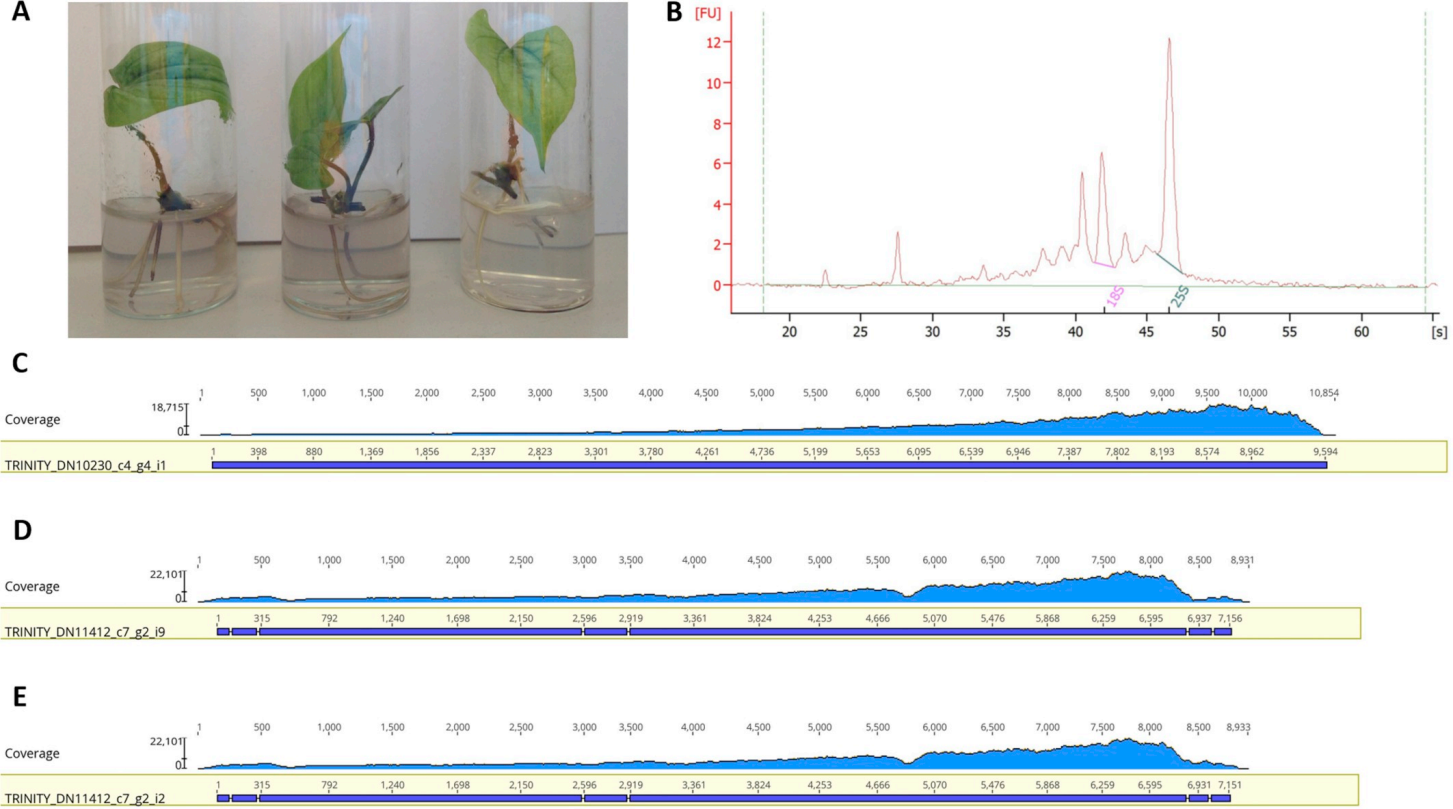
**(A**) Six-week-old tissue culture plants of the *D. rotundata* cv. Makakusa used for NGS-based virus detection. (**B**) Agilent 2100 Bioanalyzer electropherogram with RIN value 7.5. Raw RNA-seq reads were mapped to Trinity-assembled transcripts using Geneious software [60]. Contig TRINITY_DN10230_c4_g4_i1 (**C**) showed high sequence similarity (>83%) to YMV (GenBank U42596) in BLAST searches and >337,000 reads (0.88% of total reads) mapped to this contig. Contigs TRINITY_DN11412_c7_g2_i9 (**D**) and TRINITY_DN11412_c7_g2_i2 (**E**) showed high sequence similarity (88–89%) to DBRTV3 (GenBank MF476845) and >338,000 reads (0.88% and 0.89% of total reads, respectively) mapped to each of these contigs.

Following the establishment of a standardised and robust *in vitro* propagation methodology for *D. alata* and *D. rotundata* genotypes, we decided to test NGS-based virus detection in a selected yam landrace as a case study for a combined approach of virus diagnostics by NGS in yam tissue culture. For this, leaves of three *D. rotundata* (cv. Makakusa) plantlets were pooled (Fig. 3A) and high-quality total RNA was extracted (Fig. 3B) for Illumina RNA sequencing. Over 38 million reads were generated for the Makakusa yam sample and assembled using the Trinity pipeline. The RNA-seq assembled transcripts were mapped to a custom-made BLAST database containing complete YMV and badnavirus genomes publicly available from the NCBI GenBank. This approach resulted in three transcripts, of which two mapped to the DBRTV3 genome ([31]; GenBank MF476845) and one mapped to the YMV genome ([15]; GenBank U42596), indicating the presence of a mixed infection with a DBRTV3-like badnavirus and a YMV Nigeria isolate (YMV-NG) in cv. Makakusa. We propose the names “Dioscorea bacilliform RT virus, isolate DBRTV3-[2RT]” and “Dioscorea bacilliform RT virus, isolate DBRTV3-[3RT]” for the two DBRTV3-like badnavirus transcripts. We reconstructed the 5′-ends of the DBRTV3-[2RT] and DBRTV3-[3RT] genomes by extending the mapped contigs with the raw RNA-seq reads using the Geneious [60] iterative assembler with ten iterations. Two single contigs of 7453 and 7448 bp were recovered and represent the complete DBRTV3-[2RT] and DBRTV3-[3RT] badnavirus genomes, respectively. The raw RNA-seq reads were also re-mapped to the Trinity-assembled transcripts to get an approximate number of reads (below 1% of total reads for all three viral genomes) representing the identified virus genomes and interestingly showing a strong bias in the sequencing towards 3′-end of transcripts (Fig. 3C–E). This non-uniformity of read coverage is likely to have been caused by the use of oligo-dT beads to capture polyA tails in the library preparation technology [69,70].

### 3.3 Characterisation of members of the genera Badnavirus and Potyvirus identified in a yam landrace from Nigeria

The assembly of three full-length viral genomes derived from cv. Makakusa was achieved using Illumina HiSeq4000 RNA sequencing based on total RNA extracted from tissue culture leaves showing mild viral symptoms (Fig. 3A). New members of the genera *Badnavirus* and *Potyvirus* were detected. The complete genome sequences of DBRTV3-[2RT] (MG711311), DBRTV3-[3RT] (MG711312), and YMV-NG (MG711313) were deposited in the NCBI GenBank database.

#### 3.3.1 Badnavirus characterisation

DBRTV3-[2RT] and DBRTV3-[3RT] were determined to be 7453 and 7448 bp in length, with a GC content of 43.4% and 43.6%, respectively. BLAST searches confirmed that both complete genomes were most similar to DBRTV3 (88% and 89% sequence identity, respectively), a new member of the genus *Badnavirus* recently detected in *D. rotundata* breeding line TDr 89/02475 and classified within the monophyletic species group K5 [31]. Pairwise comparison of DBRTV3-[2RT] and DBRTV3-[3RT] revealed 99.1% sequence identity. The protein-coding regions of DBRTV3-[2RT] and DBRTV3-[3RT], including the badnavirus RT-RNaseH domain, were found to be identical and the two genomes only differ in their intergenic regions (IGR), suggesting that these genomes are likely to represent two versions of replicative transcripts of the same virus. Both genomes displayed all the hallmarks of a typical representative of the genus *Badnavirus* in the family *Caulimoviridae* and were annotated accordingly [19]. A plant cytoplasmic initiator methionine tRNA sequence within the IGR at position 1–18 designated the beginning of the viral genomes [71]. The tRNA^Met^-binding site of both sequences (5′-TGGTATCAGAGCTTGGTT-3′) possesses 17 of the 18 nucleotides complementary to the consensus sequence of the plant tRNA^Met^-binding site (3′-ACCAUAGUCUCGGUCCAA-5′). Moreover, a potential TATA-box (5′-TATATAA-3′) and a possible poly-adenylation signal (poly(A) tail) (5′-AATAAA-3′) located downstream of the putative transcription start site within the IGR were identified for both genomes.

**Fig. 4:**
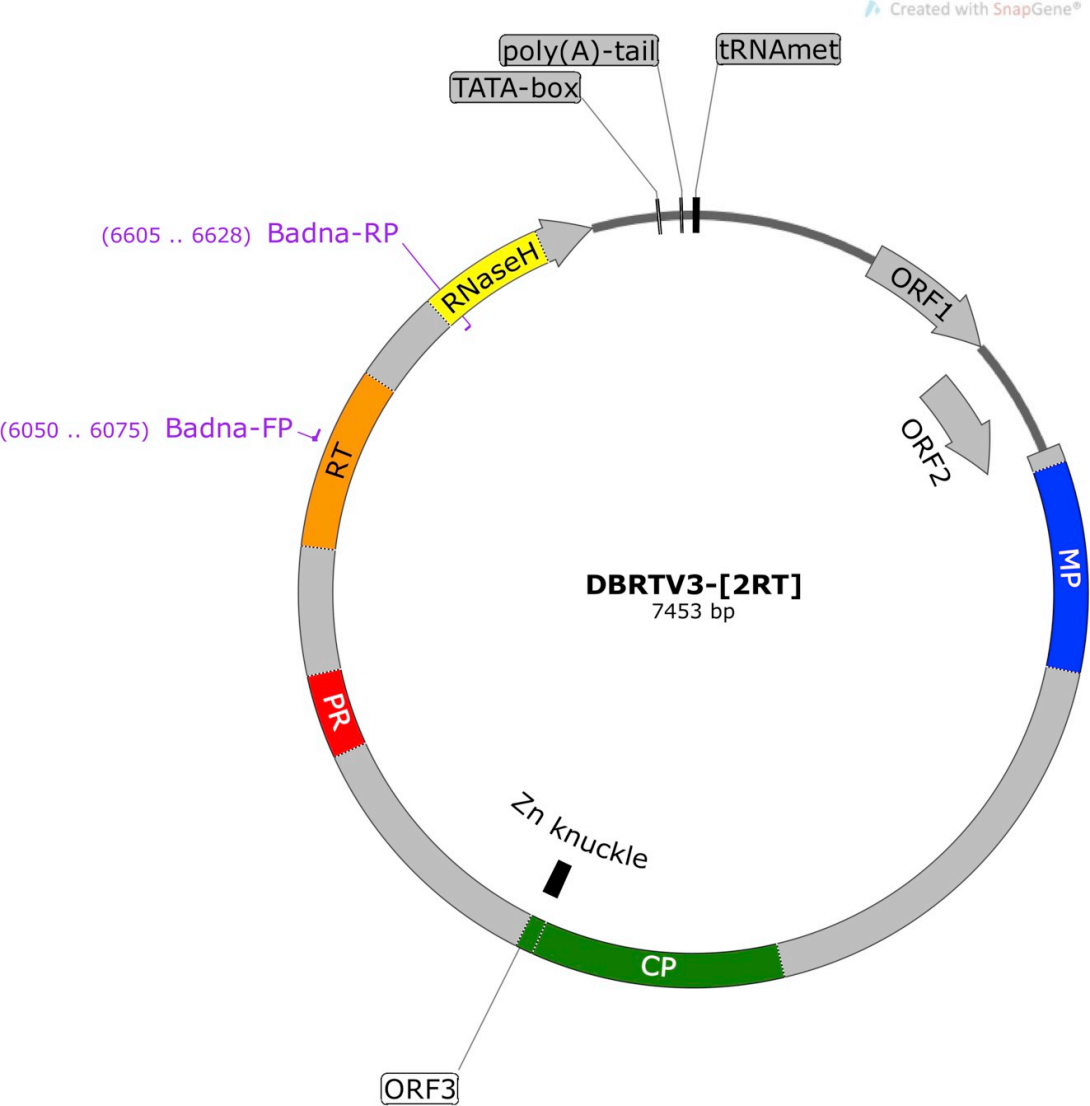
Circular representation of the Dioscorea bacilliform RT virus, isolate DBRTV3-[2RT] (GenBank accession number MG711311) genome organisation, showing the tRNA^Met^-binding site; the TATA-box; the putative poly(A) tail; open reading frame (ORF)1; ORF2; ORF3 containing putative movement protein (MP), capsid protein zinc-finger domain (CP and Zn knuckle), pepsin-like aspartate protease (PR), reverse transcriptase (RT) and RNaseH conserved motifs; and binding sites for Badna-FP/-RP primers (purple) [73], which amplify a 579-bp fragment of the RT-RNaseH domain and are used for taxonomic assessment of badnaviruses [19].

Sequence analysis of DBRTV3-[2RT] and DBRTV3-[3RT] using NCBI ORF finder revealed three closely packed ORFs, arranged in tandem on the plus strand. Consistent with the genome organisation of DBRTV3 [31], start and stop codons of ORFs 1 and 2 and ORFs 2 and 3 overlapped by the ATGA motif in a −1 translational frame relative to the preceding ORF. No internal AUG codons were identified in ORFs 1 or 2, consistent with the leaky scanning model of translation typical of members of the genus *Badnavirus* [19]. Analysis of deduced amino acid sequences predicted proteins with molecular weights of 17, 14.3, and 216 kDa encoded by ORFs 1, 2, and 3, respectively. Based on the NCBI conserved motif search, the ORF3 polyprotein of DBRTV3-[2RT] and DBRTV3-[3RT] likely encodes characteristic protein motifs of members of the family *Caulimoviridae*, including the zinc knuckle (Zn knuckle), pepsin-like aspartate protease (PR), RT, and ribonuclease H (RNaseH) [19]. The CP and movement protein (MP) described by Xu et al. [72] were also located. A circular representation of the DBRTV3-[2RT] genome is shown in Fig. 4, highlighting all features typical of genomes in the genus *Badnavirus* of family *Caulimoviridae*.

**Fig. 5:**
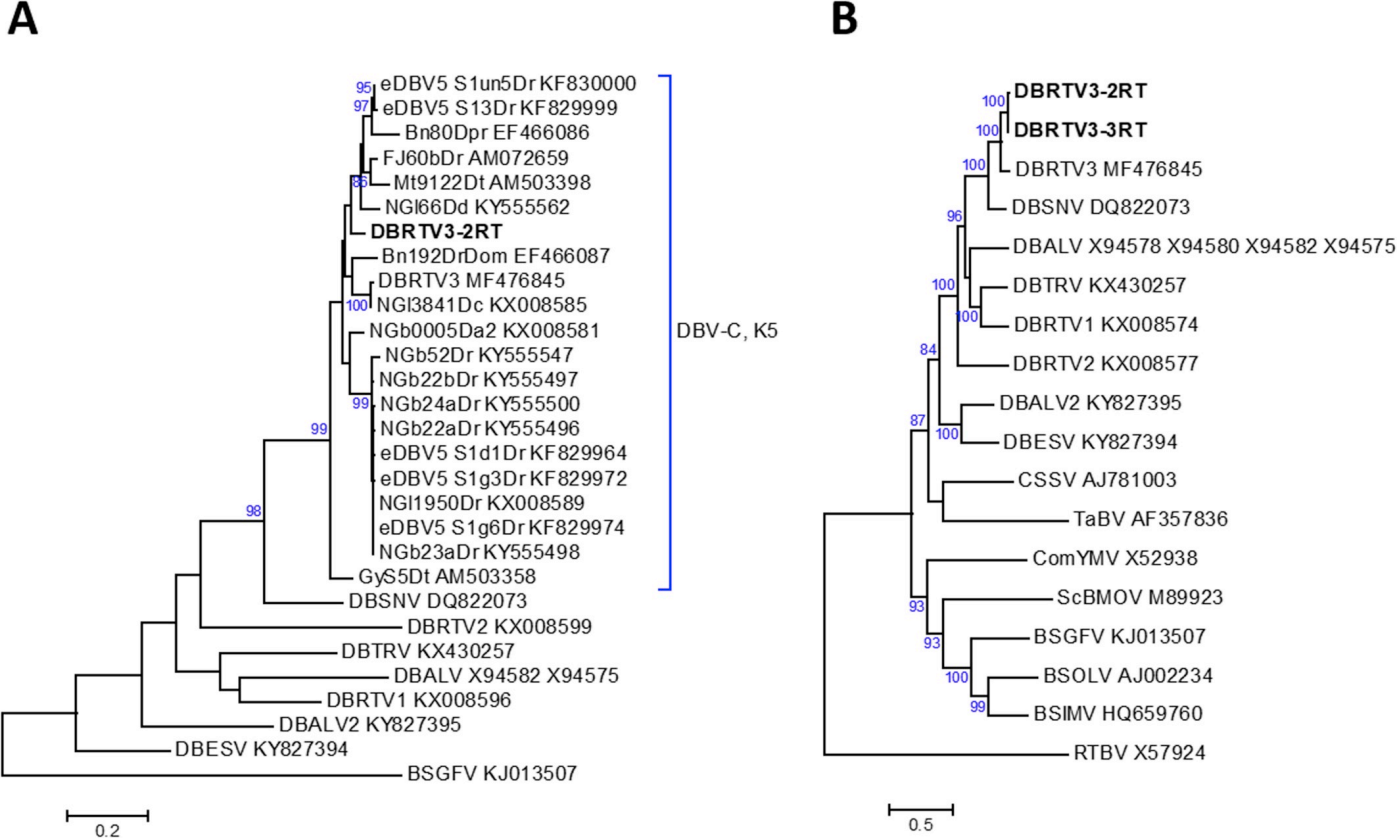
Molecular phylogenetic analysis of new members of the species *Dioscorea bacilliform virus* (DBV) belonging to the genus *Badnavirus*. Bootstrap consensus phylogenetic trees were constructed based on 528-bp partial nucleotide sequences of the badnavirus RT-RNaseH domain (**A**) or on full-length nucleotide sequences of the genomes of DBVs and other badnavirus type members (**B**). The partial RT-RNaseH sequences of eight DBV genomes and all yam badnavirus sequences with nucleotide sequence identity values above 80% relative to DBRTV3-[2RT] in similarity searches with the NCBI BLAST and consequently belonging to monophyletic species group K5 described by Kenyon et al. [24] were included in the tree presented in (**A**), and *Banana streak GF virus* (BSGFV) was used as an outgroup. *Rice tungro bacilliform virus* (RTBV) functioned as an outgroup in (**B**). GenBank accession numbers are provided, and DBRTV3-[2RT] (GenBank accession number MG711311) and DBRTV3-[3RT] (GenBank accession number MG711312) are highlighted in bold. The partial RT-RNaseH sequences of DBRTV3-[2RT] and DBRTV3-[3RT] are identical and only DBRTV3-[2RT] was included in the tree shown in (**A**). Alignments of partial RT-RNaseH sequences were performed in MEGA7 [63] using the CLUSTALW tool, and full genome alignments were done using MAFFT [64]. Evolutionary relationships were inferred using the maximum-likelihood method based on the Hasegawa–Kishino–Yano model [65], conducted in MEGA7. Bootstrap analysis was performed with 1000 replicates and the cut-off value was 80%. The trees are drawn to scale, with branch lengths measured in the number of substitutions per site.

Molecular phylogenetic analysis was undertaken based on 528-bp partial nucleotide sequences of the badnavirus RT-RNaseH domains of DBRTV3-[2RT], DBRTV3, DBALV, DBALV2, DBESV, DBRTV1, DBRTV2, DBTRV, DBSNV, and 19 additional yam badnavirus sequences available in the GenBank database with nucleotide identity values > 80% relative to DBRTV3-[2RT] in similarity searches with NCBI BLAST. DBRTV3-[2RT] is 93% identical to the sequence of an endogenous DBV described by Umber et al. ([14], eDBV5 clone S1un5Dr, GenBank KF830000) and was found to belong to the monophyletic species group K5 described by Kenyon et al. [24] (Fig. 5A). A second phylogenetic analysis was undertaken using the publicly available full-length genomes of eight DBVs and of badnavirus type members from five host plants other than yam (Fig. 5B). Yam badnaviruses form a well-supported clade in which DBRTV3, DBRTV3-[2RT], and DBRTV3-[3RT] group closely together and represent sister taxa of DBSNV in the genus *Badnavirus,* which we previously reported for DBRTV3 [31].

We recently identified a unique recombination event in DBRTV3 using recombination analysis with full-length DBV genome sequences, with DBSNV likely to be the major parent and DBALV the minor parent, providing the first evidence for recombination in yam badnaviruses [31]. Here, we repeated the same recombination analysis, replacing the DBRTV3 genome with that of DBRTV3-[2RT]. This analysis detected a total of 11 possible recombination events (Table S2). Interestingly, a very similar event (based on the location of the breakpoints) to that identified for DBRTV3 in our previous study [31] was detected here at a very high degree of confidence for DBALV instead, with all seven recombination detection methods (RDP, GENECONV, BootScan, MaxChi, Chimaera, SiScan, and 3Seq) available in RDP4 showing significant *P* values (Table S2) [66]. The putative recombination site was in the IGR of DBALV and extended into the 5′-end of ORF1. DBALV was identified as the likely recombinant, with DBRTV3-[2RT] being the virus most closely related to the minor parent (Table S2); however, the RDP4 software highlighted the possibility that DBRTV3-[2RT] is the actual recombinant and DBALV the minor parent. DBSNV was used to infer the unknown major parent. Therefore, the identified unique recombination event is in line with the previous recombination event reported for DBRTV3 [31], adding further to the field's understanding of the extent of recombination among DBV genomes, a subject that demands further research attention in the future.

#### 3.3.2 Potyvirus characterisation

**Fig. 6:**
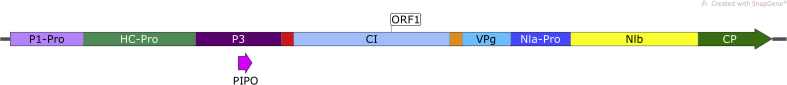
Linear representation of the genome of YMV Nigeria isolate (YMV-NG, GenBank accession number MG711313), showing P1-Pro, protein 1 protease; HC-Pro, helper component protease; P3, protein 3; PIPO, pretty interesting *Potyviridae* ORF; 6K1, 6-kDa peptide (red); CI, cytoplasmic inclusion; 6K2, 6-kDa peptide (orange); VPg, viral protein genome-linked; NIa-Pro, nuclear inclusion A protease; NIb, nuclear inclusion B RNA-dependent RNA polymerase; and CP, coat protein, according to Wylie et al. [74].

The complete nucleotide sequence of the YMV-NG single-stranded, positive-sense RNA genome was determined to be 9594 bp in length, with a GC content of 41.4%. BLAST search confirmed that the YMV-NG was most similar (85% sequence identity) to the complete genome of a YMV Ivory Coast isolate ([15]; GenBank U42596), a member of the genus *Potyvirus* collected and characterised in 1977 from naturally infected yams in the Ivory Coast [15,16]. Sequence analysis of YMV-NG using NCBI ORF finder revealed a single large ORF that putatively encodes a single polyprotein. This putative polyprotein is typically cleaved into functional proteins at semi-conserved sites by three self-encoded proteases, as is the case for most genomes of the family *Potyviridae* [74]. By comparing the YMV-NG sequence with the annotated sequence of YMV isolate Ivory Coast [15], which possesses the genome organisation of a typical member of the genus *Potyvirus* [74], and by using the NCBI conserved motif search, we identified sequences predicted to encode protein 1 protease (P1-Pro), helper component protease (HC-Pro), protein 3 (P3), 6-kDa peptide (6 K), cytoplasmic inclusion (CI), nuclear inclusion A protease (NIa-Pro), nuclear inclusion B RNA-dependent RNA polymerase (NIb), and the CP. A second small ORF was identified as pretty interesting *Potyviridae* ORF (PIPO), which is usually generated by a polymerase slippage mechanism and expressed as the *trans*-frame protein P3N-PIPO [[74], [75], [76], [77]]. A linear representation of the YMV-NG genome is shown in Fig. 6.

**Fig. 7:**
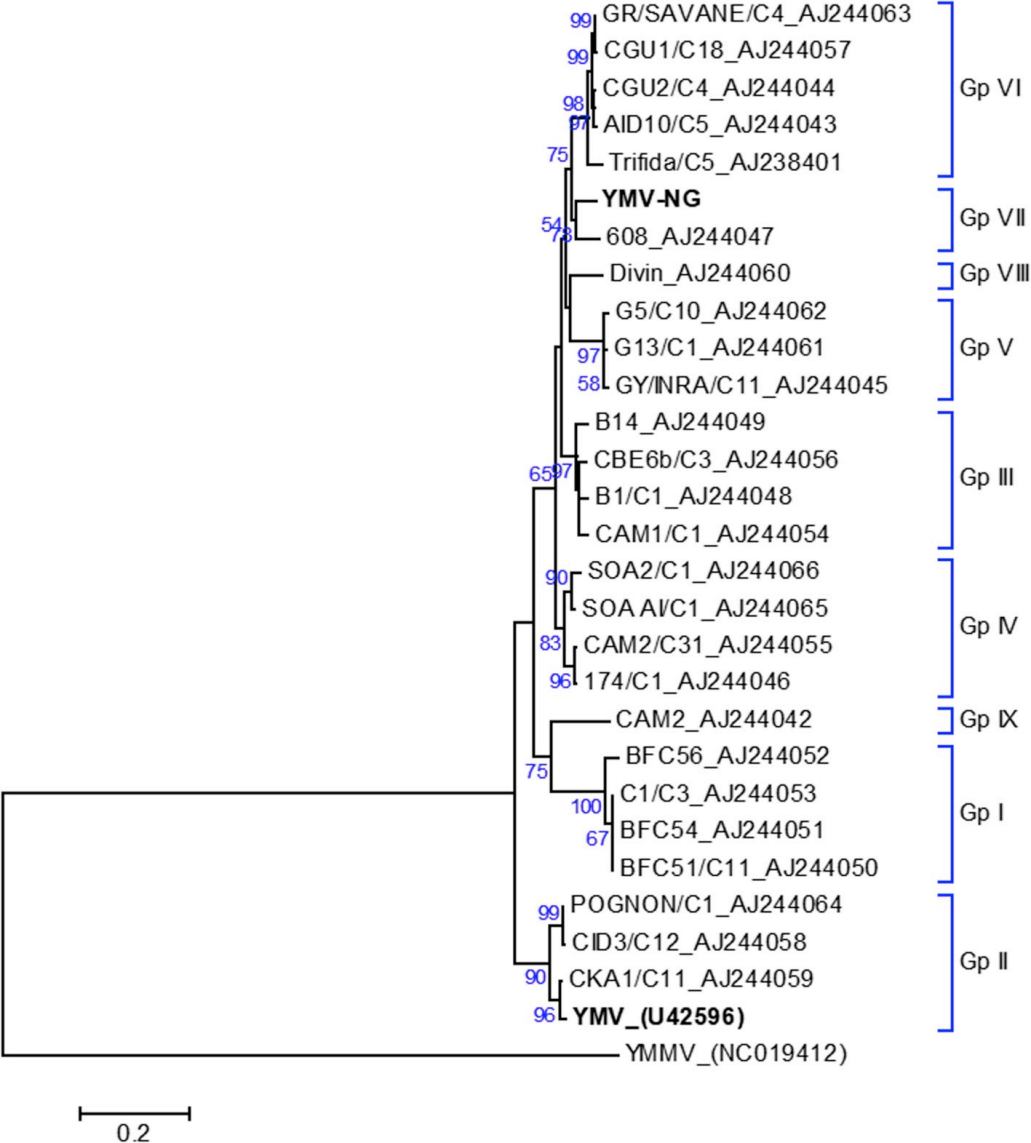
Molecular phylogenetic analysis of the NIb-CP-3′-UTR region of YMV-NG (GenBank accession number MG711313) in comparison to 26 YMV sequences and their group assignments from a phylogenetic analysis by Bousalem et al. [62]. *Yam mild mosaic virus* (YMMV) was used as an outgroup. The sequences were aligned using the CLUSTALW tool, and the evolutionary relationships were inferred using the maximum-likelihood method based on the Hasegawa–Kishino–Yano model [65], conducted in MEGA7 [63]. Bootstrap analysis was performed with 1000 replicates and the cut-off value was 50%. The tree is drawn to scale, with branch lengths reflecting the number of substitutions per site.

Molecular phylogenetic analysis was undertaken based on the NIb-CP-3′-UTR regions of YMV-NG and of 26 YMV sequences and their group assignments were compared with those described by Bousalem et al. [62]. Based on the NIb-CP-3′-UTR region, YMV-NG is most similar to a YMV partial RNA for coat protein, isolate 608 collected in Nigeria ([62], GenBank AJ244047) and is likely to belong to group VII identified in the analysis of Bousalem et al. (Fig. 7) [62]. Interestingly, Bousalem et al. [62] reported phylogenetic topological incongruent positions for YMV isolate 608, as well as for YMV isolates TRIFIDA/C5 and CAM2, and suggested that recombination events may have occurred during the evolution of YMV. We performed recombination analysis based on the NIb-CP-3′-UTR regions of all YMV sequences used in the phylogenetic analysis shown in Fig. 7, confirming a recombination event described by Bousalem et al. [62]. TRIFIDA/C5 is the likely recombinant and isolates CGU1/C18 (group VI) and G13/C1 (group V) are likely to represent the major and minor parents, respectively. No recombination events were detected for YMV-NG (data not shown). Further phylogenetic studies and recombination analyses based on complete genome sequences of YMV isolates identified in the future might shed more light on genetic diversity and evolution of the *Yam mosaic virus* species within genus *Potyvirus*, family *Potyviridae*.

### 3.4 Confirmation of virus presence using RT-PCR

**Fig. 8:**
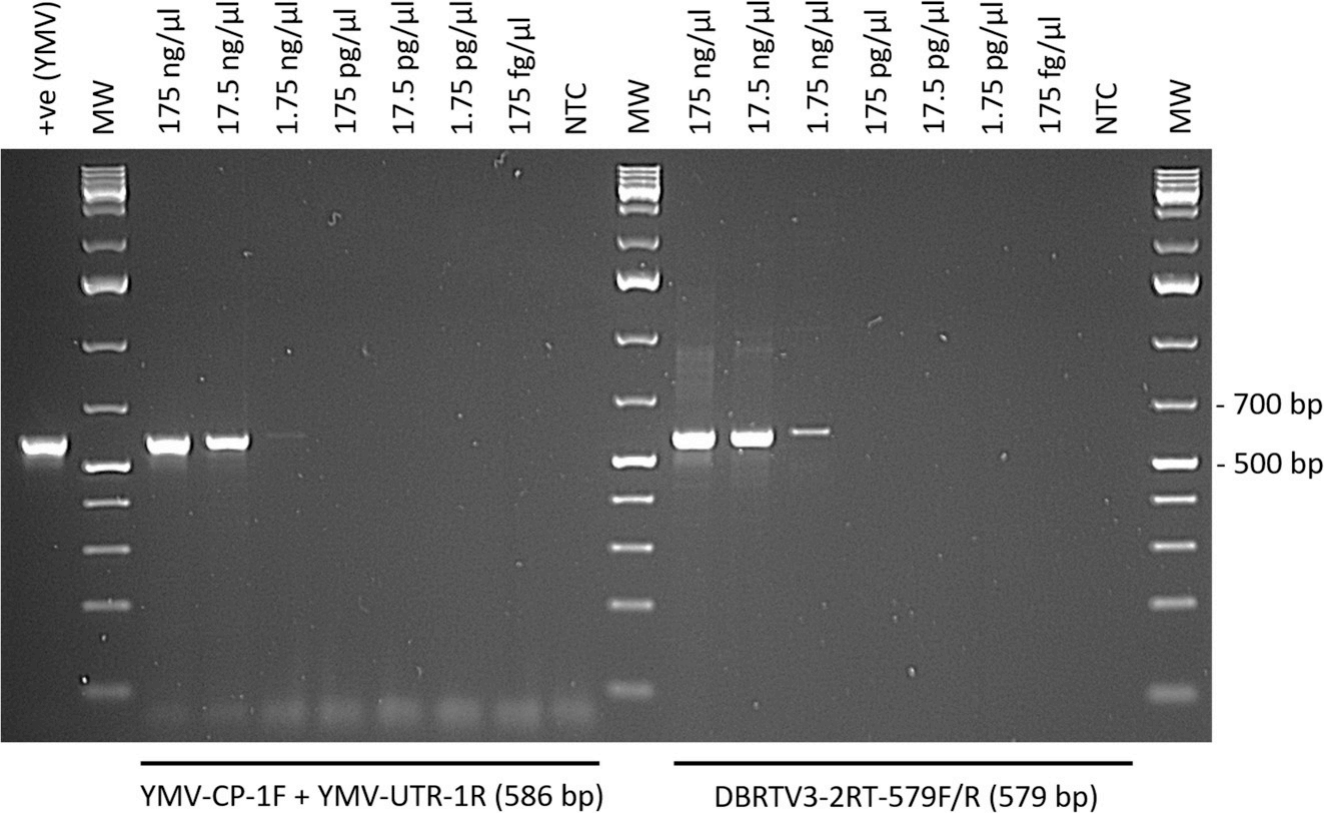
Sensitivity of one-step RT-PCR assay for the detection of YMV and DBRTV3-[2 R T] in 10-fold serially diluted total RNA from yam landrace Makakusa used for RNA-seq virus detection. MW: GeneRuler™ 1 kb Plus DNA Ladder (Thermo Scientific, UK); NTC: non-template (water) control; +ve (YMV): positive control for YMV (RNA of a YMV infected yam plant detected using the one-step RT-PCR assay described in Silva et al. [51]).

One-step RT-PCR assays were performed to confirm the mixed infection of DBRTV3-[2RT]/DBRTV3-[3RT] and YMV-NG detected by RNA-seq in cv. Makakusa grown in tissue culture (Fig. 8). One-step RT-PCR conditions for the detection of YMV were previously described by Silva et al. [51] using primers designed by Mumford and Seal [49] that target the CP and the 3′-UTR. Specific primers for DBRTV3-[2RT] were designed in this study, targeting the RT-RNaseH region used for taxonomic assessment of badnaviruses [19], and were tested using the same one-step RT-PCR conditions chosen for the YMV assay. We tested the detection limits of both one-step RT-PCR assays by making 10-fold serial dilutions of the same total RNA sample from Makakusa that was analysed by RNA-seq, starting with a total RNA concentration of 175 ng/μl. Amplification products of the expected sizes were generated in both assays and DBRTV3-[2RT]/DBRTV3-[3RT] and YMV-NG infections were confirmed by Sanger sequencing, showing identical sequences to those derived from the RNA-seq analysis (data not shown). Only weak amplification products were still detectable at 1.75 ng/μl of RNA (10^−2^ dilution).

## 4 Discussion

### 4.1 Robust yam *in vitro* culture with potential for germplasm conservation and propagation

The use of virus-free, clonally propagated planting materials is the most effective method to control the spread of viruses infecting yam [3]. Molecular diagnostic tools such as RT-RPA [51] and CP-RT-LAMP [53] have been developed for routine detection of one such virus, YMV, which is endemic in the West African ‘yam belt’ [1]. These and similar tools need to be adopted and used to verify the infection status of planting material in West Africa, where efforts to boost production of virus-free seed yam and establish sustainable seed systems are ongoing [3,53]. Research into modern yam seed production methods, including vine cutting, tissue culture, aeroponics, and TIBs, highlights the importance of an integrated multiplication scheme that combines two or more methods of seed yam production [39]. Aighewi et al. [39] further concluded that these methods need to be adopted in building and sustaining a viable seed yam production system and particularly recommends that tissue culture be included in any major seed yam production scheme due to its importance in the production and maintenance of a nucleus of clean material.

In this study, we presented a standardised *in vitro* propagation methodology for the two most important yam species, *D. alata* and *D. rotundata*. We compared two nutrient media compositions with or without the addition of AC. Different plant growth regulators present in a plant growth medium and their concentrations have a major influence on the success of *in vitro* propagation. Among plant growth regulators, auxin and cytokinins are the major determinants of root and shoot initiation in plantlets grown *in vitro*. Organogenesis (type and extent) in plant cell cultures is determined by the proportion of auxins to cytokinins [78]. Cytokinins, such as kinetin and BAP, have been proven to promote cell division, shoot proliferation, and shoot morphogenesis and to repress root formation; whereas auxins, such as NAA and dicamba, are usually used to stimulate callus production and cell growth, to initiate shoots and rooting, to induce somatic embryogenesis, and to stimulate growth from shoot apices and shoot stem culture [79]. In this study, complete plantlets (with roots and shoots) were obtained from M1 (containing kinetin) and M2 (containing NAA + BAP) media compositions. This suggests that kinetin and the combination of BAP + NAA are both capable of inducing root and shoot organogenesis from yam nodal explant material, which is in line with results observed by Poornima and Ravishankar [46] in *D. oppositifolia* and *D. pentaphylla*.

Blackening and browning of *in vitro* culture media, which is caused mainly by polyphenolic compounds, is a serious problem for the regeneration of cultured plants. This phenomenon has been observed in many woody plants [80], and yams are known to contain phenolic compounds. The AC is characterised by having a very fine network of pores with a large surface area, which generates a high adsorptive capacity, and is typically incorporated in tissue culture media to prevent browning and blackening [67]. Because of its high adsorptive capacity, AC removes inhibitory substances, such as phenolic exudates coming from cuts of the explant materials, from the culture medium [68]. It also provides a dark environment, which can provide a better environment for root development in the culture by promoting the accumulation of photosensitive auxins or co-factors at the base of the shoot [67]. We observed a significant positive effect of AC on fresh weight development in cultured yam plantlets, which supports the findings of Poornima and Ravishankar [46].

### 4.2 Value of NGS technology for identifying viruses in yam tissue culture: a case study

The ideal propagation technique for yam multiplication needs to be efficient and allow robust virus indexing. At the IITA, tissue culture is used to conserve the yam genetic resources stored at the IITA genebank (currently 5918 accessions), and selected yam accessions are cleaned of viral diseases through meristem culture [39]. Following regeneration, tissue culture plants are tested for viral infections, and negatively indexed plants are transplanted into screenhouses for establishment. Such plants are re-indexed for viruses to ensure that plants are free from virus infection. Virus-free plants are used as sources for multiplication *in vitro* or under screenhouse conditions for tuber production for international distribution [81].

However, robust virus indexing of yam *in vitro* material is challenging for two main reasons: (1) *in vitro* culture is renowned for its ability to reduce virus titres, potentially bringing certain viral infections below the detection limit of even highly sensitive diagnostic tools; and (2) standard diagnostic tests usually target only a subset of known viral species. False-negative results from routine virus indexing can potentially have dramatic consequences if, for example, infected yam germplasm is internationally distributed. Therefore, we tested whether Illumina HiSeq4000 RNA sequencing has the potential for use in robust, comprehensive, unbiased, and sensitive NGS-based virus detection in yam tissue culture material when applied without prior knowledge of the viral sequences. Here, we report an optimised protocol which includes the extraction of high-quality total RNA suitable for RNA sequencing from yam tissue culture leaves, and we show that this combined tissue culture and NGS approach allows the characterisation of novel yam mosaic and badnaviruses following a relatively simple bioinformatic pipeline. This case study is a promising step in the development of NGS-based yam virus diagnostics, and we are hopeful that this technology will be adopted in certain situations where the cost is justified to support virus-free yam propagation, distribution, and germplasm conservation.

### 4.3 Mixed infections of YMV and yam badnaviruses

Numerous full-genome sequences of known and unknown plant viruses have been discovered using NGS-based methods and subsequently validated by molecular diagnostic protocols [82]. The detection of new members of the genera *Badnavirus* and *Potyvirus* in a selected yam landrace functioned as a first case study for NGS virus diagnostics in yam. The NGS approach revealed a mixed infection with the presence of two badnavirus transcripts (DBRTV3-[2RT] and DBRTV3-[3RT]) and a novel yam mosaic virus, YMV-NG. The RNA sequencing results support previous findings obtained using a combination of RCA and PCR for the detection of DBRTV3 [31] and RPA-based diagnostic tools [51] and confirm the usefulness of NGS in plant virology. The mixed infection was further confirmed using a one-step RT-PCR approach, and the detection limit suggested low titres for both virus infections in Makakusa tissue culture.

Endogenous viral sequences can be transcriptionally active in yam species and may be functionally expressed as described for geminivirus-like elements [83]. The majority of EPRVs described to date are fragmented, rearranged, contain inactivating mutations and are therefore replication defective and consequently non-infectious. However, it remains unclear if eDBV sequences, that have been described for four distinct badnavirus species (groups K5, K8, K9, and U12) [14], are transcriptionally active and potentially infectious. Therefore, it remains remotely possible that DBRTV3-[2RT] and DBRTV3-[3RT] were assembled from eDBV5 transcripts. Future work will be performed to test for the potential existence of eDBV forms of the DBRTV3-[2RT] and DBRTV3-[3RT] sequences in yam germplasm using Southern hybridisation techniques like those described by Seal et al. [25] and Umber et al. [14], and previously discussed for DBRTV3 [31].

### 4.4 Advantages of NGS over standard molecular diagnostic tools for virus detection

Almost half of emerging plant infectious diseases are viral, according to outbreak reports [84]. In the past, the detection and characterisation of novel viruses mostly relied on electron microscopy, serological methods such as ISEM and ELISA, and nucleic acid-based methods such as PCR and microarrays [[85], [86], [87]]. Efficient routine virus diagnostic tools have become easily available because of the breakthroughs made around ELISA and PCR-based assays [88,89], and both techniques and their variants have been modified for the broad-based detection of plant viruses. In their review, Prabha et al. [55] conclude that both techniques suffer from several significant drawbacks, particularly when used in diagnosing unknown viral diseases, as all these techniques are dependent on previous knowledge about viral genome sequence information for primer design or efficient monoclonal or polyclonal antibodies targeting virus epitopes. The dependence on sequence information includes novel isothermal detection methods which are now increasingly being developed including RT-RPA and CP-RT-LAMP assays for YMV detection [51,53].

The use of degenerate primers targeting conserved sites in known viral gene sequences has led to the discovery of unknown and foreign viruses. Conserved sites are identified by sequence comparison, which means that the usefulness of degenerate primers depends entirely on how well the known sequences represent the target group, including unknown sequences [90]. According to Zheng et al. [90], sampling bias in the past has misled researchers attempting to identify conserved target sites (‘consensus decay’) to design degenerate primers targeting the genus *Potyvirus*, and regular updating of primer design is needed. The degenerate badnavirus-specific primer pair Badna-FP/-RP [73] has led to the discovery of several hundred badnavirus sequences across different plant hosts and hence is a good example of the usefulness and power of this approach. However, in the case of yam badnaviruses, the extreme heterogeneity of DBVs [26], mixed infections [27], and presence of integrated counterparts in the form of complex mixtures of eDBV sequences [33] means that there is still a need for the development of a robust diagnostic test for all episomal DBVs. Current diagnostic practices for DBV screening using the Badna-FP/-RP primer pair are likely to introduce many false positive results due to the presence of eDBV sequences in *D. cayenensis-rotundata* genomes [14,25,27,33], which cannot be distinguished from DBVs in a simple Badna-PCR. Additionally, false-negative results cannot be excluded because of sequence heterogeneity and the presence of mixed infections and potentially low titres.

Compared with routine serological and nucleic acid-based diagnostic methods, NGS technologies can provide a more comprehensive picture of the entire plant virome in a selected sample where the additional cost of NGS can be justified. The NGS enables the unbiased detection and discovery of novel viruses and their complete genomes without prior knowledge of the viral sequences. These massive parallel sequencing approaches advance our understanding of viral genome variability, evolution within the host, and virus defence mechanism in plants and are therefore extremely useful for plant virology [55,91], although the infectivity of some identified viral sequences cannot be determined from some NGS datasets. The NGS-based virus diagnostic approaches enable the characterisation of complete viral genome sequences, which can then be used for phylogenetic or recombination analysis as shown in this study. The discovery and characterisation of larger numbers of complete viral genome sequences will increase our understanding of viral evolution and the molecular interactions between plant viruses and their hosts.

Whereas the future points to adoption of NGS approaches in routine plant virus discovery and characterisation, several challenges remain to be addressed; for example, dependency of available classification algorithms on homology despite high diversity in viral sequences and limited reference viral genomes in public databases. Secondly, the analysis tools are less intuitive to use, prompting specialised bioinformatics expertise and expensive computational resources. This has become a major bottleneck in making NGS approaches affordable despite the massive reduction in the cost of sequencing over the past decade.

## 5 Conclusions

We present a case study for sensitive NGS-based virus detection in yam plants grown using a robust tissue culture methodology. *In vitro* culture media compositions containing different plant growth hormones were compared, and a standardised protocol for yam tissue culture, high-quality total RNA extraction, and NGS analysis was developed. Illumina HiSeq4000 RNA sequencing from leaf material grown in tissue culture was utilised to identify novel members of the genera *Badnavirus* and *Potyvirus,* highlighting the utility of NGS-based virus diagnostics in yam. Two badnavirus isolates, DBRTV3-[2RT] and DBRTV3-[3RT], as well as a novel *Yam mosaic virus* isolate, YMV-NG, were detected in a cv. Makakusa sample from Nigeria, and complete genomes were assembled and characterised for these three viral isolates. The YMV and badnavirus infections were confirmed in RNA extracted from tissue-cultured plant material using one-step RT-PCR. This study presents a promising first step towards developing a robust *in vitro* propagation and NGS-based virus detection protocol, and confirms the value of NGS in safe movement of germplasm.

## Conflicts of interest

None.

## Appendix A Supplementary data

Data Profile Data Profile
