# Supplementary material for: Tissue culture and next-generation sequencing: A combined approach for detecting yam (Dioscorea spp.) viruses
Source: Physiol Mol Plant Pathol. 2019 Jan;105:54–66. doi: 10.1016/j.pmpp.2018.06.003 (PMC6472605; doi:10.1016/j.pmpp.2018.06.003)
Supplement: Fig. S1 [file mmc2.docx]

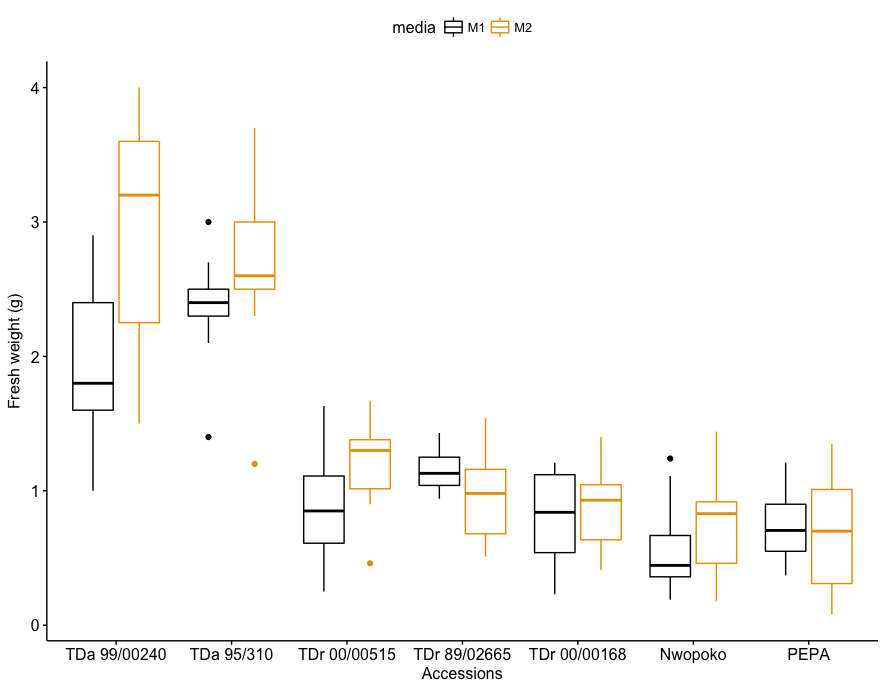


**Figure S1.** Comparison between fresh weight development of individual *D. alata* (TDa) and *D. rotundata* (TDr) accessions grown as yam *in vitro* culture on different media. Fresh weight measurements were taken at 70 days after *in vitro* culture growth. The fresh weight data (g) are expressed as mean ± SE. M1 and M2 denote different *in vitro* culture media compositions described in Table 1.
